# Cytokine/chemokine profiles in squamous cell carcinoma correlate with precancerous and cancerous disease stage

**DOI:** 10.1038/s41598-019-54435-0

**Published:** 2019-11-28

**Authors:** Zewen K. Tuong, Andrew Lewandowski, Jennifer A. Bridge, Jazmina L. G. Cruz, Miko Yamada, Duncan Lambie, Richard Lewandowski, Raymond J. Steptoe, Graham R. Leggatt, Fiona Simpson, Ian H. Frazer, H. Peter Soyer, James W. Wells

**Affiliations:** 1 The University of Queensland Diamantina Institute, Faculty of Medicine, The University of Queensland, Translational Research Institute, Brisbane, QLD Australia; 2 Dermatology Research Centre, The University of Queensland, The University of Queensland Diamantina Institute, Translational Research Institute, Brisbane, QLD Australia; 3 Diabetes Center, University of California, San Francisco, CA United States; 4 Future Industries Institute, University of South Australia, Adelaide, SA Australia; 5 IQ Pathology, South Brisbane, QLD Australia; 6 Greenslopes Private Hospital, Greenslopes, QLD Australia

**Keywords:** Squamous cell carcinoma, Tumour biomarkers, Chemokines, Chemokines, Cancer epidemiology

## Abstract

Actinic Keratosis (AK), Intraepidermal Carcinoma (IEC), and Squamous Cell Carcinoma (SCC) are generally considered to be advancing stages of the same disease spectrum. However, while AK often regress spontaneously, and IEC often regress in response to immune-activating treatments, SCC typically do not regress. Therefore, it is vital to define whether fundamental immunological changes occur during progression to SCC. Here we show that proinflammatory cytokine expression, chemokine expression, and immune cell infiltration density change during progression to SCC. Our findings suggest a switch from predominantly proinflammatory cytokine production to chemokine production is a key feature of progression from precancer to cancer. Together, these observations propose a model that can underpin current research and open new avenues of exploration into the clinical significance of these profiles with respect to immunotherapeutic or other treatment outcomes.

## Introduction

Actinic Keratosis (AK) and Intraepidermal Carcinoma (IEC or squamous cell carcinoma (SCC) *in situ*), are generally considered to be early, premalignant stages of cutaneous SCC^1^. While AK and IEC undergo a high rate of spontaneous regression, estimated by Marks *et al*. to be as high as 25.9% for AK^2^, patients with these lesions are often treated with topical immunomodulatory agents such as imiquimod to reduce the risk of progression to SCC^3,4^. SCC, by contrast, displays a low clinical response rate to imiquimod, which is, therefore, not recommended for therapy of invasive SCC^5^. Immunological alterations to the local environment likely occur during progression to SCC and this could provide an explanation for such disparate treatment responses. However, the differences in the immune environment associated with each disease stage remain poorly defined.

It has been appreciated for more than 40 years that SCC are highly immunogenic tumours that are quickly rejected when adoptively transferred from UV-treated mice into immune competent mice, but not rejected when adoptively transferred into thymectomized mice^6^. This suggests a key role for T cells in the control of SCC, which is further supported by the observation that patients receiving T cell-suppressive drugs to prevent organ transplant rejection suffer a 65–250 fold increased risk of SCC development compared with the general population^7^. CD8 T cells and gamma-delta T cells are known to migrate into SCC^8,9^, and in a recent Phase I/II study Migden *et al*. reported complete- and partial responses in approximately 50% of advanced SCC patients treated with the PD-1 checkpoint blockade antibody Cemiplimab^10^, further underscoring an important role for immune surveillance in the control of SCC.

We recently examined the abundance of T cells in AK, IEC, and SCC lesions taken from immunocompetent patients and discovered that there is a decreased abundance of CD8 T cells in SCC compared with IEC^11^. This observation appears to contrast with studies examining two chemokines important for T cell infiltration into the skin, where CXCL10 expression (defined by microarray^12^), and CCL27 expression (defined by immunohistochemistry^13^), were both increased in SCC lesions compared with IEC lesions. However, despite a common association with immune infiltrates that express the chemokine receptor CXCR3^14^, very little is understood about the chemotactic environment in IEC and SCC lesions that could underlie alterations in T cell trafficking. Understanding changes that modulate immune cell homeostasis and/or their functional characteristics, particularly T cells, in patient lesions, could facilitate the development of prognostic biomarkers or targeted therapies to enhance SCC treatment outcomes.

We hypothesized that analyzing patterns of cytokine/chemokine secretion would provide novel insights into the immune-microenvironment of the progressive stages present within the SCC disease spectrum. Here, we analyzed cytokine and chemokine abundance within patient lesions at distinct, histopathologically well-defined disease stages across SCC progression. By characterizing the cytokine profiles of AK, IEC, and SCC disease stages we sought to determine whether an alteration in the expression levels of key proinflammatory cytokines, or a change in chemokine secretion bias, may provide an indication for the reduced numbers of CD8 T cells that infiltrate into SCC.

## Results

### SCC lesions are thicker than AK and IEC lesions, but contain a similar density of inflammatory infiltrate

**Figure 1:**
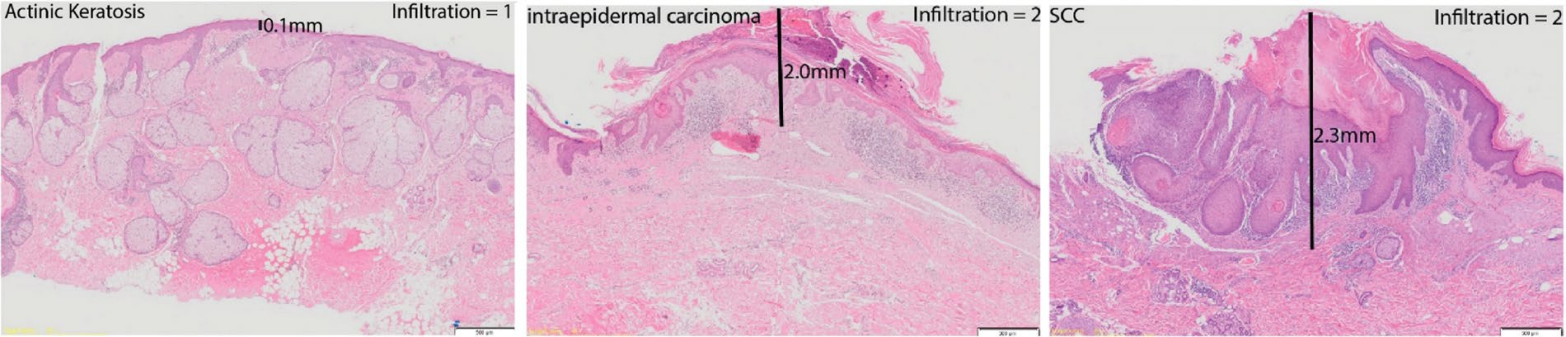
Representative photomicrographs of patient lesions showing lesion thickness measurements and examples of cellular infiltration gradings. Images presented are H&E stains, scale bar = 500 µm. Lesion thickness and immune cell infiltration was assessed as described in Materials and Methods. SCC: Squamous Cell Carcinoma.

**Figure 2:**
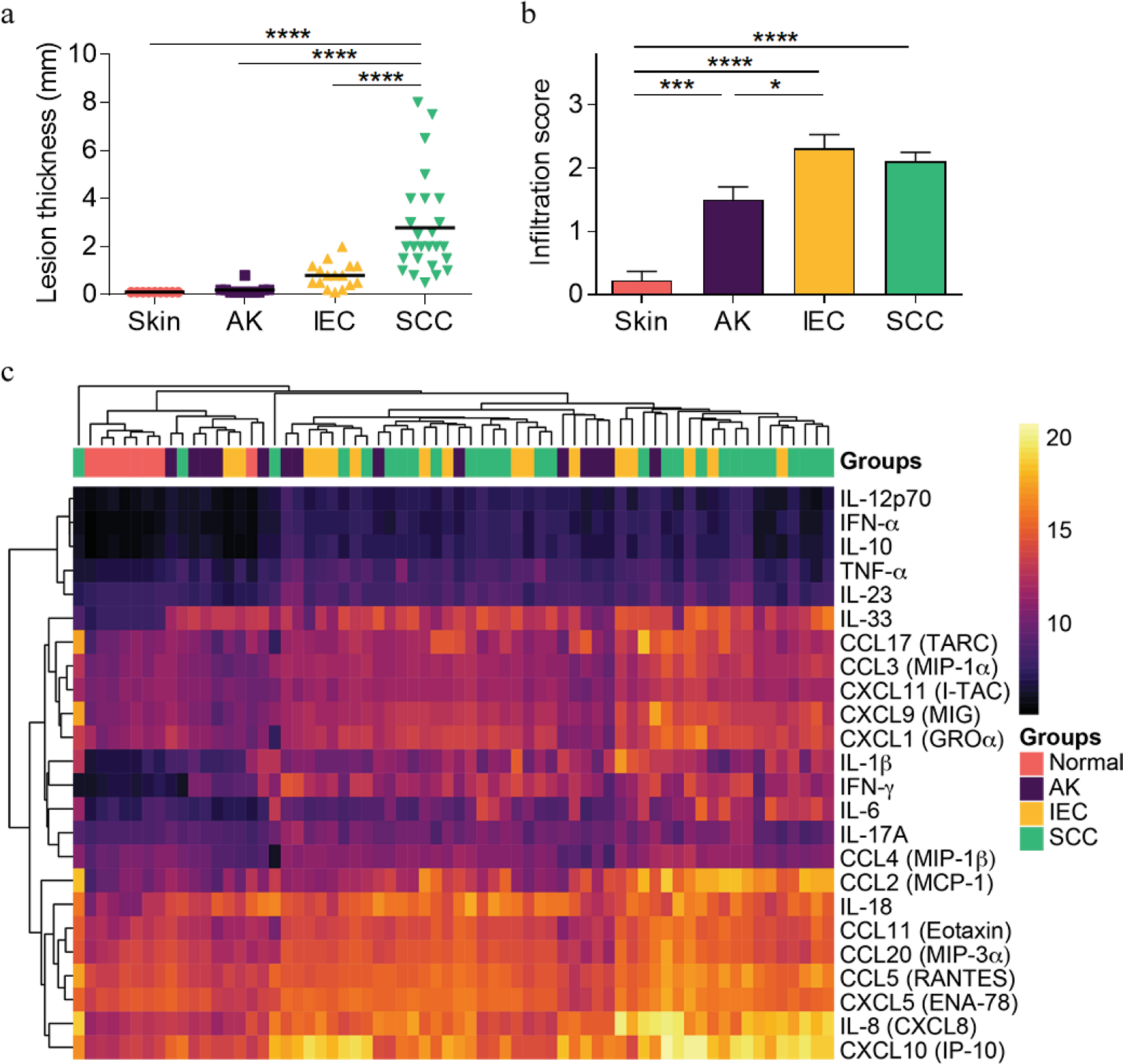
Comparison of lesion thickness and density of immune infiltrate with lesion diagnosis. Lesion thickness and immune cell infiltrate scores were determined as outlined in Fig. 1 and compared against lesion diagnosis. (**a**) Lesion thickness vs. Lesion type. (**b**) Infiltration score vs. Lesion subtype. One-way ANOVA with post-hoc Tukey’s multiple comparisons test. (**c**) Heat map color corresponds to the Log-transformed concentrations. The spectrum of black to purple to orange to yellow corresponds to increasing gradient of chemokine/cytokine concentrations. Unsupervised hierarchical clustering was performed across samples (columns) and cytokines/chemokines abundance (rows) and are ordered according to similarity of the expression profile by samples. Normal = peritumoural skin.

The depth or ‘thickness’ of skin and AK, IEC, and SCC lesions as well as the degree of inflammatory infiltrate density was determined in 67 biopsies, including 58 histopathologically well-defined stages of SCC disease (Fig. 1). As expected, lesion thickness increased with advancing stages of SCC (Fig. 2a), in line with the restriction of AK and IEC lesions to the epidermis and the extension of SCC into the dermis. The degree of inflammatory infiltrate density also increased significantly from skin to AK to IEC, but not between AK and SCC, nor between IEC and SCC (Fig. 2b). Together, the data indicate that in spite of their increased thickness when compared to AK and IEC lesions, SCC lesions do not contain increased densities of inflammatory cells.

### Individual cytokine/chemokine expression levels within SCC disease stages do not correlate with similarities or differences in the density of inflammatory infiltrate

To further investigate the immune environment within defined stages of SCC disease, we examined the abundance of 24 cytokines and chemokines within patient lesions. Excess skin and lesion biopsy tissue not required for diagnostic purposes was homogenized and assessed for the presence of chemokines by cytometric bead array. Unsupervised clustering of the cytokine/chemokine data resulted in the ordering of normal samples clustered on the left and then followed by AK, IEC and SCC. In general, normal skin samples displayed a lower abundance of all cytokines and chemokines measured when compared to lesions (Fig. 2c). Notably, CXCL10, IL-8, CXCL5, CCL5, CCL20, CCL11 and IL-18 were the most highly abundant chemokines and cytokines detected across the samples (Fig. 2c).

**Figure 3:**
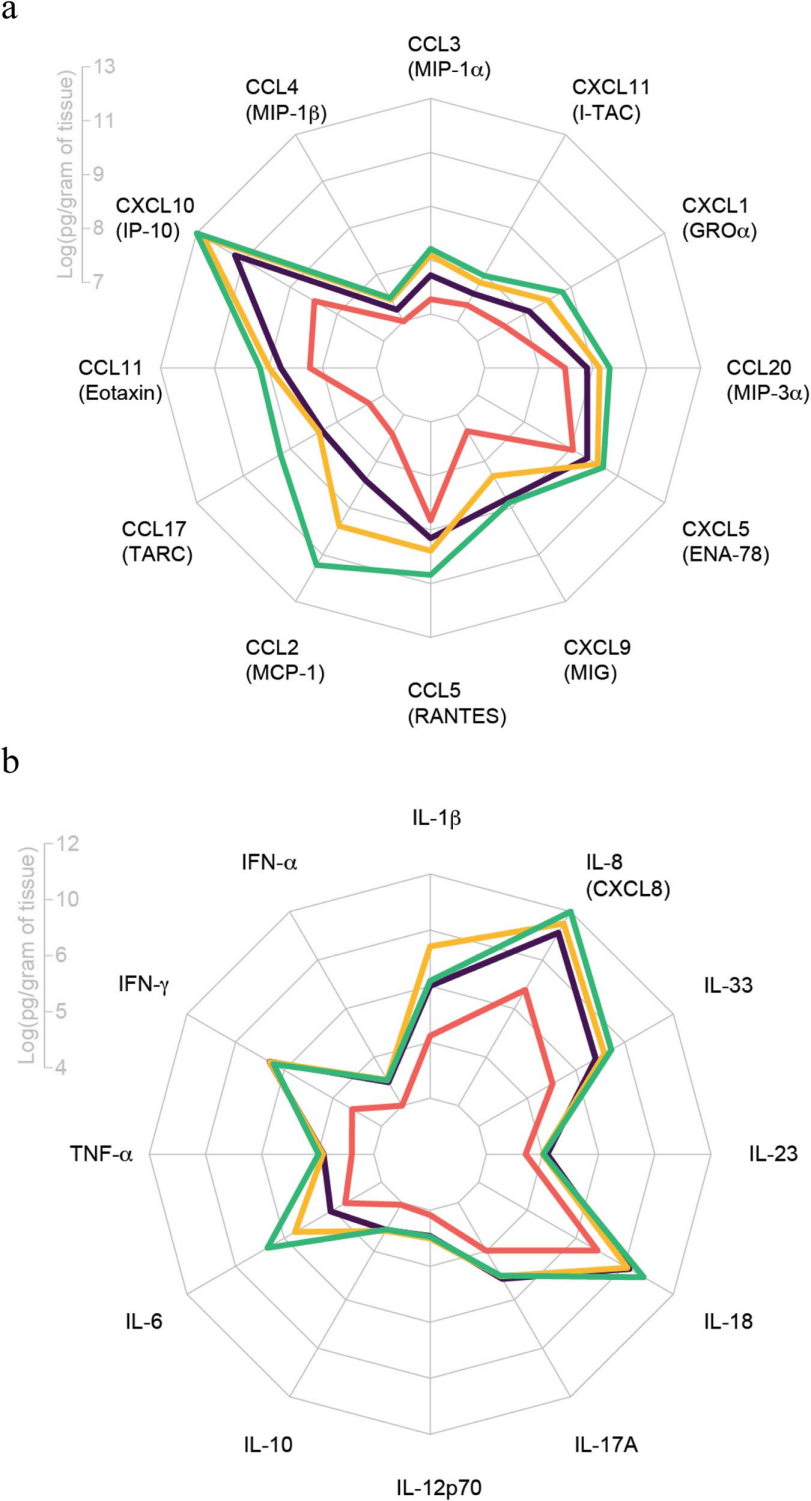
Increases in chemokine and proinflammatory cytokine abundance with disease stage. Homogenized patient skin and lesions were analyzed by cytometric bead array to determine chemokine and proinflammatory cytokine content. Radar chart of mean log-abundance values of chemokines (**a**) or proinflammatory cytokines (**b**). The axis length at each radius ranges from the minimum to maximum magnitude of log-abundance values across the 12 analytes and the axis labels mark the log-abundance values at quartile intervals (0%, 25%, 50%, 75%, 100%).

We focused first on the abundance of 12 chemokines known for their capacity to induce immune cell migration. The mean log-abundance values across the chemokines are summarized in the radar plot (Fig. 3a). CXCL10 was the most abundant chemokine in lesions and was the only chemokine significantly increased in AK compared to normal skin (Fig. 3a and Supplementary Fig. 1). When IEC was compared with normal skin, increases were also detected in CCL3, CCL2, and CXCL9 (Supplementary Fig. 1). No differences were noted when IEC was compared to AK for any of the chemokines examined. CCL5 was the only chemokine increased significantly in SCC compared to IEC, however, when SCC was compared to AK, CCL3, CCL2, CXCL9, CCL5, CCL11, CXCL5, CCL20, CXCL1, and CXCL11 were all significantly increased (Supplementary Fig. 1). All chemokines examined were significantly increased in SCC when compared with normal skin.

We further assessed the abundance of 12 inflammatory cytokines within our homogenized samples by cytometric bead array. IL-8 and IL-18 were the most abundant cytokines measured in the cohort (Fig. 3b). Furthermore, the levels of IL-18 and IL-8 were significantly elevated in IEC and SCC lesions compared to normal skin, and IL-6 was significantly elevated in SCC compared to normal skin (Supplementary Fig. 2). Levels of IL-1β, IFN-α, IFN-γ, TNF-α, IL-10, IL-12p70, IL-17A, and IL-33 were all significantly elevated in AK, IEC, and SCC lesions compared to normal skin (Supplementary Fig. 2). Interestingly however, only IL-6 expression differed *between* stages of SCC disease, with a significant elevation in expression in SCC compared to AK (Supplementary Fig. 2). Levels of IL-23 did not change between sample groups.

In summary, we observed that most chemokines and cytokines expressed within AK, IEC, and SCC lesions were increased when compared to normal skin but there was no clear distinction between the degrees of inflammatory infiltrate or disease stage.

### Correlations between cytokine or chemokine abundance and lesion thickness

**Figure 4:**
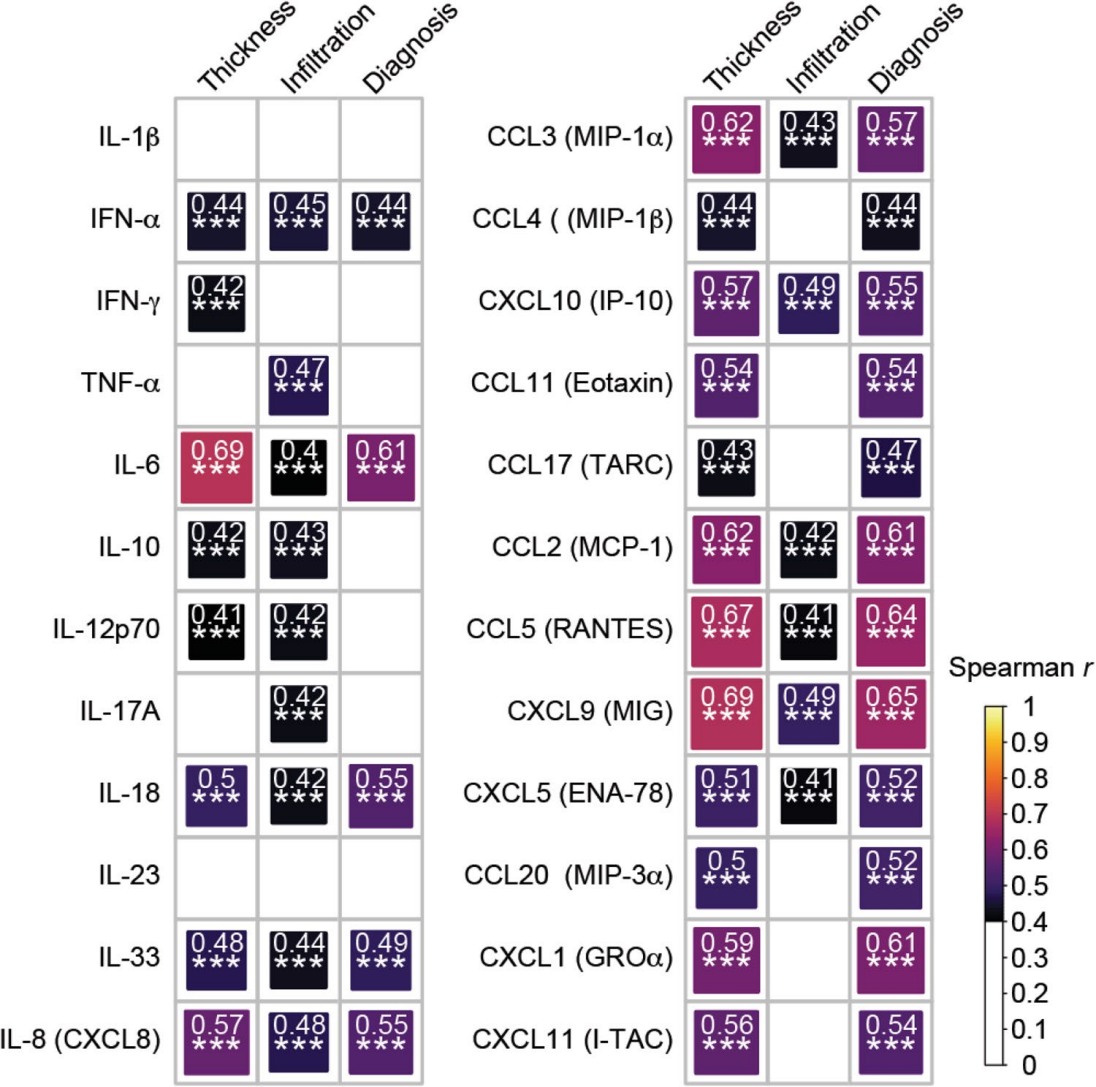
Spearman correlation of cytokine/chemokine abundance with lesion thickness, infiltration and diagnosis. Spearman correlation r values are plotted as a heatmap with the area of each square corresponding to the strength of the correlation. Correlations that reached r > 0.4 and achieved a two-tailed t-test p-value of < 0.05 are shown. The correlation and statistical testing was performed using the rcorr function embedded in the Hmisc R package.

To determine how levels of cytokine or chemokine expression might be impacted by lesion thickness, we performed a correlation analysis. As shown in Supplementary Table 1, we observed a strong correlation (r > 0.5) between expression of IL-6, CXCL9, CCL5, CCL3, CCL2, CXCL1, IL-8, CXCL10, CXCL11, CCL11, and CXCL5 and lesion thickness. A moderate correlation (r = 0.3–0.5) was found in the expression of IL-18, CCL20, IL-33, IFN-α, CCL4, CCL17, IL-10, IFN-γ, IL-12p70, IL-17A, IL-1β, and TNF-α with lesion thickness, while the correlation was weak (r < 0.3) between IL-23 and lesion thickness. The same analysis was repeated to examine the correlation between the cytokine/chemokine abundance with infiltration score and lesion diagnosis. We observed that some cytokines are significantly correlated with r > 0.4 across all three comparisons, including IFN-α, IL-6, IL-18, IL-33, IL-8, CCL3, CXCL10, CCL2, CCL5, CXCL9, and CXCL5 (Fig. 4). Among them, IL-6, CCL2, CCL5 and CXCL9 achieved r > 0.6 in at least two of the correlation assessments (Supplementary Table 1).

### PCA reveals a divergent pattern of chemokines and cytokines in SCC lesions

**Figure 5:**
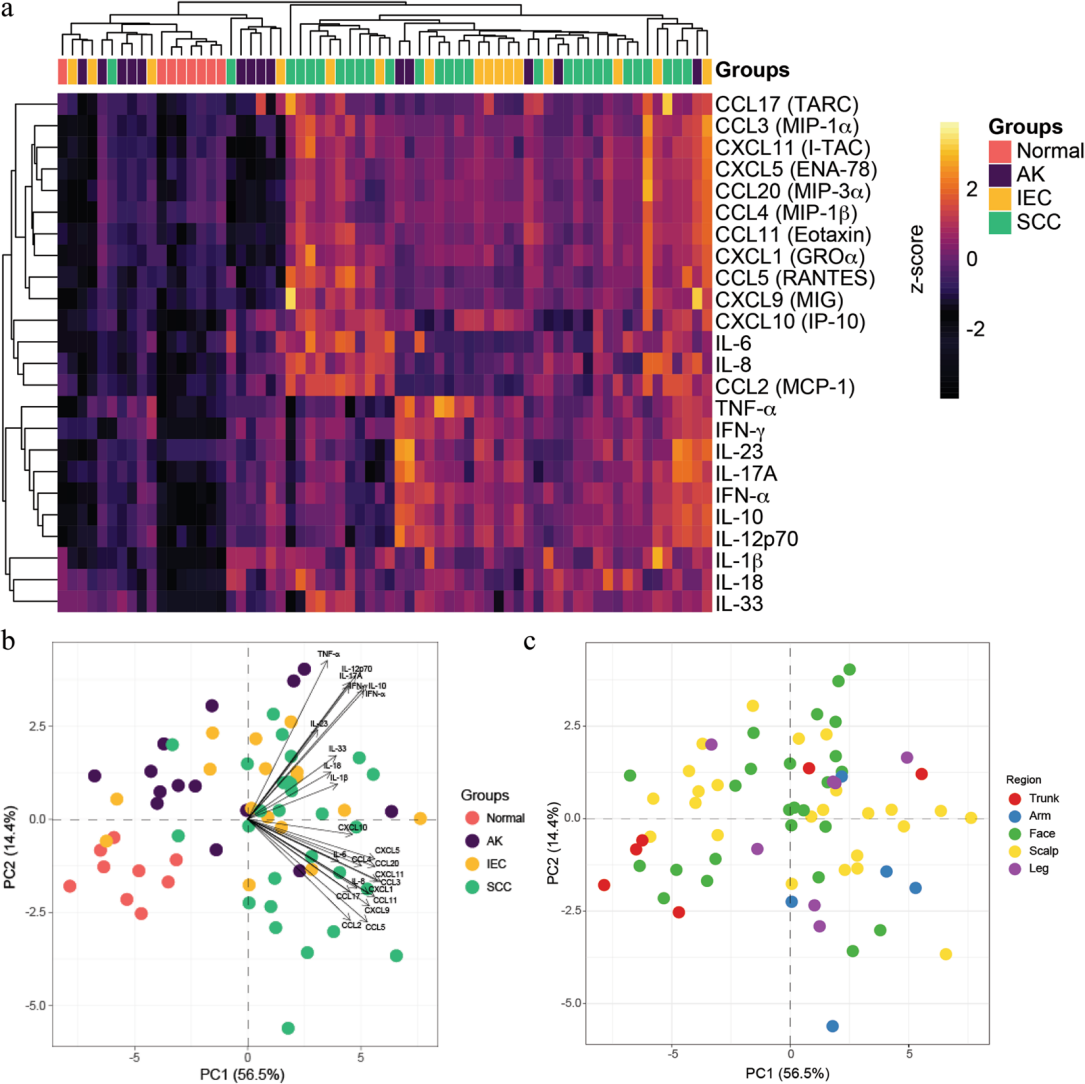
Cytokine and chemokine associations with disease stage. (**a**) Heat map color corresponds to the scaled Log-transformed abundance values represented along a z-scale; Log-transformed abundance values for each chemokine and cytokine were centered to 0 and the heat represents the standard deviations away from the center. The spectrum of black to purple to orange to yellow corresponds to increasing gradient of chemokine/cytokine concentrations. Positive values indicate higher expression while negative values indicate lower expression. Unsupervised hierarchical clustering was performed across samples (columns) and scaled cytokines/chemokines abundance (rows) and are ordered according to similarity of the expression profile by samples. (**b**,**c**) PCA analysis using (**b**) lesion type and (**c**) lesion location presented as a biplot. Each point represents a sample and is colored according to the diagnosis (**b**) or sample origin (**c**). The positions on the plot corresponds to their corresponding coordinates along PC1/PC2 axis. The arrows in (**b**) indicate the direction and contribution/weight of each variable (chemokine/cytokine) where longer arrows indicate higher variance and smaller cosine (angles between arrows) indicates the higher degree of correlation between variables. The arrows point in the direction of increasing concentrations.

When we examined the scaled Log-transformed abundance values for both chemokines and cytokines as an expression heat map, it reflected the earlier conclusion that there is an overall relative increase in all chemokines and cytokines in lesional skin compared to normal skin (Fig. 5a). However, it also suggests that there may be some subtle separation effects in the lesions that were not immediately obvious. For example, hierarchical clustering showed that some lesions samples can be roughly divided into high chemokine and/or high cytokine expressing lesions, or alternatively into low expressing lesions (Fig. 5a). Therefore, in order to explore this ‘structure’ of the data further, we employed principal component analysis (PCA) to examine how the lesions are separated based on the overall variance (in chemokines and/or cytokines) in the data. The analysis was performed on the scaled Log-transformed abundance values to normalize the distribution and reduce the bias associated with extremely highly- and lowly- expressed chemokines and cytokines. The first two principal components (PC) of the data are displayed, which accounts for the largest (PC1) and second largest (PC2) source of variation between the samples (Fig. 5b).

The largest source of variance separating the sample groups, as shown in distribution across PC1, can be largely attributed to lesion progression (Fig. 5b). The effect associated with the second largest source of variance is not immediately obvious; however, overlaying the contribution of the variables (chemokines and cytokines, denoted by arrows in Fig. 5b), we can observe that the increase in proinflammatory cytokines TNF-α, IL-17, IL-12p70, IFN-γ, IL-10 and IFN-α is associated with displacement of samples towards the upper-right quadrant (Fig. 5b). Similarly, increased expression of the chemokines CXCL10, CXCL5, CXCL20, CXCL11, CCL3, CXCL1, CCL17, CCL11, CXCL9, CCL5, CCL2 and IL-8 (CXCL8) is associated with displacement of samples towards the lower-right quadrant (Fig. 5b). In contrast, normal skin samples, in which chemokines and cytokines are generally less abundant (Fig. 5a), are located in the lower-left quadrant (Fig. 5b). Interestingly, AK samples are generally located in the upper-left quadrant (Fig. 5b). The component loadings (weighting of the different cytokines) also suggest that the cytokine profile in the upper right quadrant (Fig. 5b) is reminiscent of proinflammatory cytokine expression, whereas the lower right quadrant (Fig. 5b) contains predominantly chemokine expression. No clear associations could be visualized between cytokine profile and the original biopsy site (Fig. 5c).

**Figure 6:**
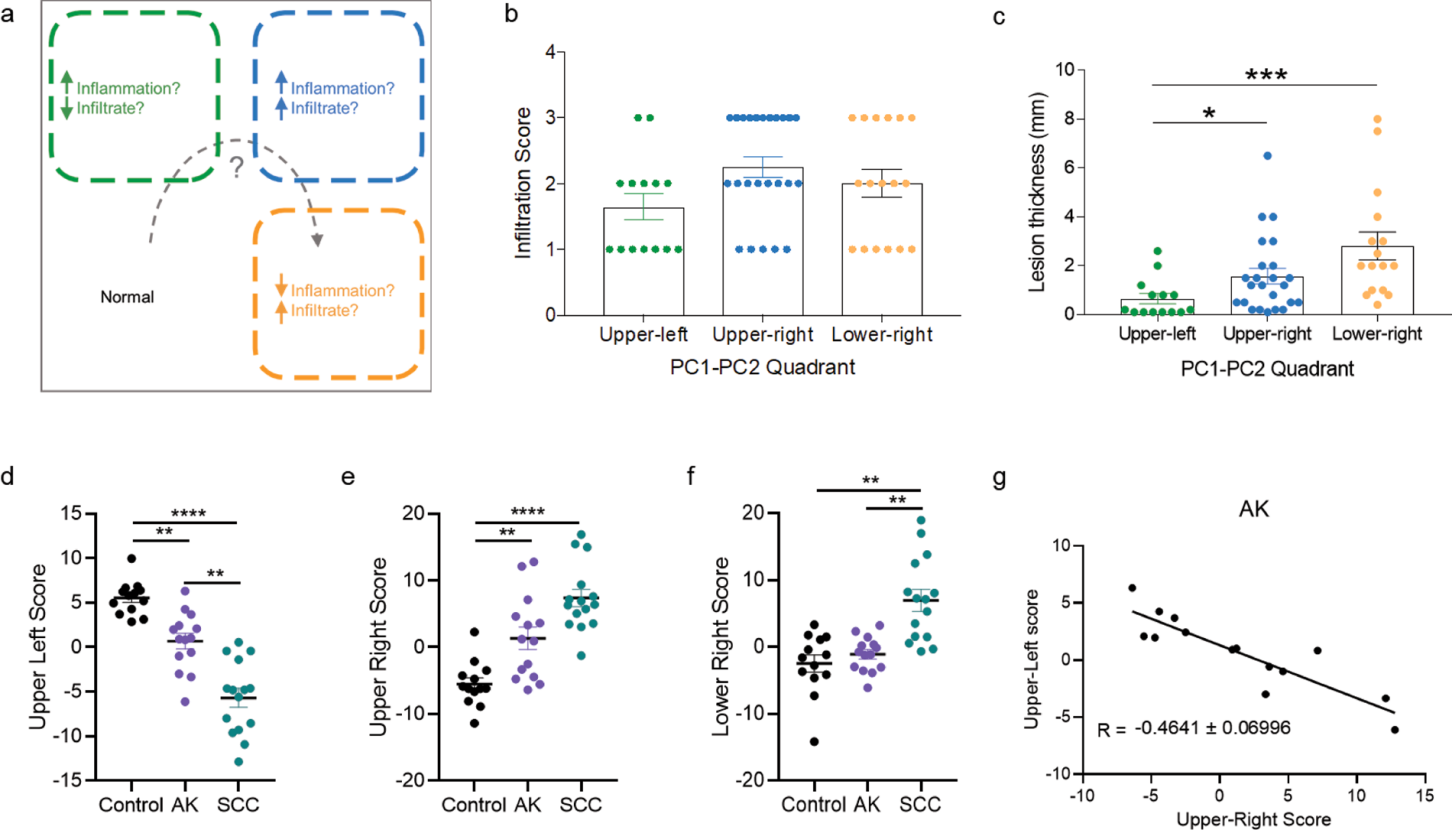
Model describing the emergence of divergent subtypes of SCC. (**a**) Illustration of the proposed state of samples according to the distribution in PC1-PC2 dimensions. Normal: skin. (**b**) Immune cell infiltration scores and (**c**) Average lesion thickness re-grouped according to quadrant locations. (**c**) Kruskal-Wallis test (P = 0.0007) followed by Dunn’s multiple comparisons test. (**d**) Upper-left, (**e**) upper-right and (**f**) lower-right scores (logit; log-odds) for logistic regression analysis for samples in each diagnosis category. One-way ANOVA with Tukey’s multiple correction was performed where **P < 0.01; ****P < 0.0001. (**g**) X-Y plot of upper-right scores versus upper-left scores for AK showing negative correlation for samples scoring highly in either signature.

The analysis supports a hypothetical model where each quadrant (empirically set at the 0 intersect of PC1 and PC2 in Fig. 5b) represents a different ‘state’ – the lower-left quadrant represents a ‘normal’ state; the upper -left quadrant represents an increased inflammatory state with relatively reduced immune cell infiltrate; the upper-right quadrant represents a state with a relative increase in both inflammatory markers and immune cell infiltrate; and lastly the lower-right quadrant represents a state with relatively increased immune infiltrate but reduced pro-inflammatory cytokine profile (Fig. 6a). In support of this hypothesis, we re-plotted the pathology measurements of lesion thickness and immune cell infiltrate scores and grouped samples contained in the upper-left (9 AK, 3 IEC, 2 SCC), upper-right (3 AK, 9 IEC, 12 SCC), or lower-right (1 AK, 3 IEC, 13 SCC) quadrants irrespective of their disease staging. The analysis showed that the infiltration score in the upper-right and lower-right quadrants were marginally increased compared to the upper-left quadrant, but this did not attain statistical significance (Fig. 6b). Lesion thickness was significantly increased in samples assigned to the upper-right and lower-right quadrants compared to those from the upper-left quadrant (Fig. 6c).

Finally, we tested whether the cytokine/chemokine profiles found herein could be observed in other datasets of skin SCC progression. We trained a logistic regression model for the three quadrant signatures (upper-left, upper-right and lower-right) derived from our cytokine/chemokine profiling data and scored it against a publically available AK/SCC microarray dataset (GSE32628)^15^. We found that normal samples scored higher for the upper-left signature as this signature models decreases in CCL3, CCL4, CCL11, CCL2, CCL5, CXCL5, CXCL1, CXCL11 and increases in IFN-γ and IL-33 (Fig. 6d). AK and SCC scored higher for the upper-right signature compared to normal samples as this signature models increases in IL-1β, IFN-α, IFN-γ, TNF-α, IL-10, IL-12A, IL-17, IL-23A, CCL4, CXCL10, CCL11 and CXCL5 (Fig. 6e). SCC was the only group that showed a higher score in the lower-right signature as this signature models increases in IL-6, IL-8, CCL3, CXCL10, CCL17, CCL2, CCL5, CXCL9, CXCL1, CXCL11, and decreases in IFN-α, TNF-α and IL-23A (Fig. 6f). Although AK showed enrichment of both upper-left and upper-right signature, there was a negative correlation of the scores within AK i.e. samples scoring higher in the upper left signature tended to score lower in the upper-right signature and vice versa (Fig. 6g). This analysis is consistent with our observations that the chemokine signature is not present in AK and that there is a dichotomous cytokine/chemokine profile within AK.

In summary, the analysis suggests that (i) up-regulation of proinflammatory cytokines is a feature in AK that separates it from normal skin, (ii) the altered chemokine profile of IEC and SCC is more pronounced than that of AK, and (iii) SCC disease stages are separable into distinct cohorts based on immune cell infiltration, proinflammatory cytokine expression, and chemokine expression, with a corresponding difference in lesion thickness.

## Discussion

Non-melanoma skin malignancy typically presents as a spectrum of progressive dysplasia manifesting from benign AK through to IEC, and eventually, aggressive cutaneous SCC. It has been well reported that increasing levels of inflammation are associated with disease progression, with SCC lesions demonstrating the highest signs of keratinization, inflammation, increased CD4^+^:CD8^+^ T cell ratio, and vascularization across the disease spectrum^11,16,17^. Surprisingly, however, SCC lesions contain reduced numbers of CD8 T cells compared to precancerous IEC lesions^11^. This latter observation led us to examine the chemokine and proinflammatory cytokine environment within lesions at histopathologically defined disease stages. Here, we interrogated the cytokine and chemokine profile of biopsies obtained from 37 subjects and found that, while the inflammatory molecules measured were globally increased in lesions compared to normal skin controls, the lesions could be separated based on their cytokine and chemokine profile. Importantly, this observation was made in patients that were deemed as immunocompetent (i.e. patients were not on immunosuppressive medication). This observation has the potential to guide appropriate selection of immunotherapeutic or other treatment options for non-melanoma skin malignancy.

The lack of clinically distinct- and limited molecular differences defined to date has made delineating the continuum of AK, IEC, and SCC challenging. Indeed, the clinical segregation of IEC from AK as a distinct intermediary disease stage has been called into question^18,19^. Further, our analysis of 24 cytokines and chemokines within AK and IEC lesions, when compared individually, did not show any distinct differences between these two lesion types. Although global increases in cytokine/chemokines correlated well with lesion thickness, they did not correlate well with immune cell infiltration, and indeed while immune cell infiltration was significantly more prominent in IEC and SCC lesions, there was not much difference between them (as per pathological assessment, Fig. 2b). However, by performing a PCA to interrogate the variance between samples in a hypothesis-free situation, our analysis suggested a model (Fig. 6a) in which SCC disease stages can be separated based on the inflammatory state of the lesion, i.e. a split in cytokine versus chemokine enrichment, even when the detected levels of all the measurements are globally increased. We found that the PCA approach was particularly useful, and important, in this aspect as it reduces the dimensionality of multi-parameter data to enable an unbiased data-driven approach to understand the subtle differences that underlie or occur during SCC progression, which is not immediately obvious when looking at individual analytes. This analysis technique is frequently applied to analysis of high-dimensional datasets such as those obtained in transcriptomics and proteomics studies, but it has also been applied to the analysis of multiplex cytokine assays^20^. However, we acknowledge a major caveat in our analysis in that it does not provide an explanation/indication for non-discriminate cases nor as to why there is such a large variability in cytokine/chemokine expression patterns in similarly staged lesions.

The divergence in cytokine patterns is reminiscent of a recent report describing two subclasses of SCC with distinct origins, identified by differences to keratinocyte gene methylation patterns. The two distinct subclasses differ in their methylation patterns which present as either a stem cell-like profile or a keratinocyte-like profile^21^. The distinctive methylomes were also conserved when keratin genes alone were investigated, reinforcing that two subclasses of AK/SCC exist^21^. Whether the immune epigenome in SCC progression was similarly affected or not is unknown as this was not assessed in the study; however, it is increasingly evident that epigenetic modification of immune cells primes immune cell fate and function, as exemplified by regulation of NK cell and CD8 T cell differentiation and acquisition of immunological ‘memory’ in a virus infection model, for example^22^. Whether attributable to a difference in origin, or features of disease progression such as epigenetic modifications, we speculate that a relative reduction in proinflammatory cytokine expression in thicker lesions may be an underlying factor that promotes lesion growth, potentially by restricting appropriate inflammatory signals received by infiltrating immune cells. Likewise, a switch towards chemokine enrichment may also have adverse impact in the form of recruitment of immunosuppressive cells. For example, accumulation of CCR5^+^ myeloid-derived suppressor cells in a mouse model of melanoma is associated with local up-regulation of CCR5 ligands (CCL3/MIP-1α, CCL4/MIP-1β and CCL5/RANTES)^23^. Similarly, regulatory T cells have also been reported to home to a mouse model of cutaneous SCC via CCR5 signalling^24^. In both examples, the CCR5 chemokine signalling pathway is paramount to the immunosuppressive tumour microenvironment^23,24^. The up-regulation of chemokines in cutaneous SCC may be due to intrinsic repair responses activated by keratinocytes^25^ when faced with a pathological insult; however in tumours, this repair response may be chronically activated inappropriately, and ultimately contribute to immunosuppression. Indeed, up-regulation of chemokines during progression of skin malignancy appears to be a consistent feature observed in human SCC compared to normal skin^26^. This has been corroborated in a mouse model of HPV-associated skin premalignancy, particularly for the CXCL9/10-CXCR3 signalling pathway^27–30^. In this study, we confirmed the relevance of up-regulated CCR5 ligands, as well as multiple other chemokines, including CXCL9, −10 and −11, in human skin samples of well-defined SCC progression stages.

A limitation present in our study is that our correlation analyses were performed under the assumption that there is a linear progression from AK to IEC to SCC. However, our observations of cytokine divergence were independent of this analysis, revealing a subtle dimorphic enrichment of proinflammatory cytokines or chemokines in higher grade lesions. Altogether, our findings support a model (Fig. 6a) where AK prominently differ from surrounding skin through the up-regulation of proinflammatory cytokines, and two subsets of SCC can be differentiated from AK based on their proinflammatory cytokine/chemokine expression profile. Going forward, it is likely that single-cell transcriptomics and proteomics, and perhaps epigenomics applications in the study of skin cancer may shed light on the relatively unknown heterogenous processes that contribute to skin cancer development, including understanding how cutaneous SCC may arise^31^, as well as the complex immune interplay within the tumour microenvironment and how it influences the success of immunotherapies^32^.

Here, we demonstrate through application of chemokine and cytokine profiling that SCC disease stages can be distinguished with a high degree of confidence, providing new insights into the mechanisms of and/or the immune consequences of SCC progression. The clinical significance of these profiles with respect to immunotherapeutic or other treatment outcomes, will be an important focus for future investigations.

## Materials and Methods

### Ethics statement

This study was carried out in accordance with the recommendations of the National Statement on Ethical Conduct in Human Research (2007), Greenslopes Research and Ethics Committee. The protocol was approved by the Greenslopes Research and Ethics Committee. All subjects gave written informed consent in accordance with the Declaration of Helsinki.

### Patient samples

All patient samples were collected from outpatients at The Greenslopes Private Hospital, Greenslopes. A total of 67 biopsies including 58 lesions suspicious for keratinocytic malignancy from 37 immunocompetent patients were included in this study (13 females and 24 males). Patient lesions had not been treated with medication previously, the mean age of lesion donors was 78.45 (45–96), and the vast majority of lesions were derived from moderately or severely sun-damaged sites on the scalp, face, arm, or calf. In total, 9 ‘normal’ skin samples (photodamaged, peritumoural), 14 AK lesions, 16 IEC lesions, and 28 SCC lesions were analysed. Following lesion removal, fresh, excess tissue not required for diagnostic purposes was de-identified prior to analysis at The University of Queensland Diamantina Institute, Translational Research Institute, Brisbane. Specimen pathology including H&E staining was performed by IQ Pathology (South Brisbane, Queensland) and diagnosis correlated to sample analysis post-hoc by H.P.S. Analysis data were not made available to H.P.S prior to correlation.

### Cytokine and chemokine analysis

4 mm punch biopsies were taken from excised specimens and transferred into cold PBS containing a broad-spectrum non-toxic protease inhibitor cocktail (EDTA-free “Mini-complete protease inhibitor cocktail”, Roche). Samples were kept on ice, and within 2 hours of harvest they were weighed, homogenized in 500 µl of PBS containing Mini-complete protease inhibitor cocktail, and frozen at −80 °C. Homogenates were analyzed for cytokine and chemokine content using the LEGENDplex Human Inflammation Panel and the LEGENDplex Human Proinflammatory Chemokine Panel, respectively, (both from Biolegend, San Diego, USA) as per the manufacturer’s instructions. Analysis was performed by flow cytometry using a Gallios Flow Cytometer (Beckman Coulter, Lane Cove, Australia) and data analyzed using LEGENDplex Data Analysis Software (Biolegend). All measurements were normalized to starting lesion weight (pg per gram wet tissue weight).

### Measurement of tumour thickness and density of cellular infiltrate

Lesion H&E histopathology slides were scanned at 20X magnification using the Olympus VS120 slide scanner, and analyzed using Olympus Olyvia software V2.9. Lesion thickness was measured from the granular layer to the deepest part of the tumour. Immune cell infiltration was measured visually by histopathological assessment of H&E paraffin mounted slides of specimens (H.P.S & M.Y.), and scored from 0 to 3 for presence and density of cellular infiltrate (number of cells with the appearance of lymphocytes per cross-section examined).

### Statistical analysis

Unless otherwise stated, statistical analysis was carried out using GraphPad Prism version 7.03 for Windows GraphPad Software, San Diego, CA, USA. Statistical tests are as indicated in figure legends. The correlations of cytokine abundance with lesion thickness were analyzed using a two-tailed Spearman correlation analysis following confirmation that the data were not normally distributed using a D’Agostino & Pearson omnibus normality test. Diagnosis was converted to an ordinal scale where 1 to 4 is assigned to normal, AK, IEC and SCC respectively. Relationship strengths were described as follows: (r) > 0.5 = strong; 0.3 to 0.5 = moderate; < 0.3 = weak. *P* values of *p* < 0.05 (*) were considered significant. *p* < 0.01 (**), *p* < 0.001 (***) are indicated. Principal Component Analysis (PCA) was performed using Log-transformed cytokine and chemokine bead array data. The transformed data was inspected for normality using Q-Q plots. Spearman’s correlations and PCA was performed in the R statistical package version 3.5.0. Elastic-net regularized (alpha = 0.5) logistic regression was performed using the R package *Glmnet*^33^ with 10-fold cross validation. The models were trained using cytokine/chemokine expression data of samples found in the respective quadrants. The models were tested on the expression data extracted from GSE32628^15^ using the *GEO2R* web tool.

## Supplementary information

Supplementary File

## Data Availability

The datasets generated during and/or analysed during the current study are available from the corresponding author on reasonable request.
