# Supplementary material for: Cytokine/chemokine profiles in squamous cell carcinoma correlate with precancerous and cancerous disease stage
Source: Sci Rep. 2019 Nov 28;9:17754. doi: 10.1038/s41598-019-54435-0 (PMC6882799; doi:10.1038/s41598-019-54435-0)
Supplement: Supplementary file 1 — Supplementary File [file 41598_2019_54435_MOESM1_ESM.docx]

**Cytokine/chemokine profiles in squamous cell carcinoma correlate with precancerous and cancerous disease stage**

**Zewen K. Tuong^1#^, Andrew Lewandowski^1,2#^, Jennifer A. Bridge^1,3#^, Jazmina L. G. Cruz^1#^, Miko Yamada^2,4#^, Duncan Lambie^5^, Richard Lewandowski^6^, Raymond J. Steptoe^1^, Graham R. Leggatt^1^, Fiona Simpson^1^, Ian H. Frazer^1^, H. Peter Soyer^1,2*^ and James W. Wells^1,2*^.**

**Supplementary Fig.1. Increases in chemokine abundance with disease stage.** Homogenized patient skin and lesions were analyzed by flow cytometry using the LEGENDplex Human Proinflammatory Chemokine Panel to determine chemokine content. Bar charts are ordered in accordance with overall abundance in SCC (high to low) and normalized per gram of starting tissue weight. One-way ANOVA with post-hoc Tukey’s multiple comparisons test.

**Supplementary Fig.2. Cytokine quantification within skin, and AK, IEC, and SCC lesions.** Homogenized patient skin and lesions were analyzed by flow cytometry using the LEGENDplex Human Inflammation Panel to determine proinflammatory cytokine content. Bar charts are ordered in accordance with overall abundance in SCC (high to low) and normalized per gram of starting tissue weight. One-way ANOVA with post-hoc Tukey’s multiple comparisons test.

**Supplementary Table 1. Correlation between cytokine abundance and lesion thickness, infiltration, and diagnosis**

|  | Correlation coefficient (r) | | | *P* value | | |
| --- | --- | --- | --- | --- | --- | --- |
| Cytokine/  Chemokine | **Thickness** | **Infiltration** | **Diagnosis** | **Thickness** | **Infiltration** | **Diagnosis** |
| IL-6 | 0.69130085 | 0.40335136 | 0.60703904 | 1.83E-10 | 0.00071321 | 5.16E-08 |
| CXCL9 | 0.68967699 | 0.49250622 | 0.65483491 | 2.10E-10 | 2.30E-05 | 1.84E-09 |
| CCL5 | 0.67047602 | 0.41432138 | 0.64275464 | 9.98E-10 | 0.00049104 | 4.51E-09 |
| CCL3 | 0.6234501 | 0.43012071 | 0.57491602 | 2.90E-08 | 0.00028039 | 3.62E-07 |
| CCL2 | 0.62222123 | 0.42060781 | 0.61090473 | 3.15E-08 | 0.00039419 | 4.02E-08 |
| CXCL1 | 0.5936063 | 0.35872188 | 0.60651381 | 1.87E-07 | 0.00287401 | 5.33E-08 |
| IL-8 | 0.57475643 | 0.47506243 | 0.54932685 | 5.53E-07 | 4.86E-05 | 1.48E-06 |
| CXCL10 | 0.56874378 | 0.48636551 | 0.54867556 | 7.70E-07 | 3.01E-05 | 1.53E-06 |
| CXCL11 | 0.56128283 | 0.38311822 | 0.54132235 | 1.15E-06 | 0.00137434 | 2.25E-06 |
| CCL11 | 0.54098466 | 0.35191506 | 0.54073409 | 3.28E-06 | 0.00349605 | 2.32E-06 |
| CXCL5 | 0.50778344 | 0.4122606 | 0.51959887 | 1.58E-05 | 0.00052721 | 6.61E-06 |
| IL-18 | 0.49894002 | 0.42146126 | 0.54815033 | 2.33E-05 | 0.00038249 | 1.57E-06 |
| CCL20 | 0.49678951 | 0.36196918 | 0.51520795 | 2.56E-05 | 0.00261364 | 8.15E-06 |
| IL-33 | 0.47903684 | 0.43694836 | 0.48713969 | 5.42E-05 | 0.00021821 | 2.91E-05 |
| IFN-α | 0.44484814 | 0.45008324 | 0.44331456 | 0.00020504 | 0.00013268 | 0.00017189 |
| CCL4 | 0.44403621 | 0.36423812 | 0.43648658 | 0.00021127 | 0.00244443 | 0.00022198 |
| CCL17 | 0.42643715 | 0.3741049 | 0.46713896 | 0.00039699 | 0.00181689 | 6.74E-05 |
| IL-10 | 0.42457191 | 0.43372188 | 0.38896383 | 0.00042359 | 0.00024582 | 0.00114189 |
| IFN-γ | 0.42016117 | 0.3771024 | 0.38633768 | 0.00049309 | 0.00165726 | 0.00124153 |
| IL-12p70 | 0.40833337 | 0.4218984 | 0.37892144 | 0.00073363 | 0.00037661 | 0.00156664 |
| IL-17A | 0.38156173 | 0.42248125 | 0.33242814 | 0.00171194 | 0.00036891 | 0.0059874 |
| IL-1β | 0.34539807 | 0.34310989 | 0.3678286 | 0.00483324 | 0.0044767 | 0.0021966 |
| TNF-α | 0.32404659 | 0.47112821 | 0.37322796 | 0.00845539 | 5.73E-05 | 0.00186614 |
| IL-23 | 0.25415505 | 0.29539965 | 0.24341228 | 0.04105397 | 0.01522958 | 0.04715741 |
